# Food Intake Is Influenced by Sensory Sensitivity

**DOI:** 10.1371/journal.pone.0043622

**Published:** 2012-08-20

**Authors:** Katherine R. Naish, Gillian Harris

**Affiliations:** School of Psychology, University of Birmingham, Birmingham, United Kingdom; University of Missouri-Kansas City, United States of America

## Abstract

Wide availability of highly palatable foods is often blamed for the rising incidence of obesity. As palatability is largely determined by the sensory properties of food, this study investigated how sensitivity to these properties affects how much we eat. Forty females were classified as either high or low in sensory sensitivity based on their scores on a self-report measure of sensory processing (the Adult Sensory Profile), and their intake of chocolate during the experiment was measured. Food intake was significantly higher for high-sensitivity compared to low-sensitivity individuals. Furthermore, individual scores of sensory sensitivity were positively correlated with self-reported emotional eating. These data could indicate that individuals who are more sensitive to the sensory properties of food have a heightened perception of palatability, which, in turn, leads to a greater food intake.

## Introduction

The rising incidence of obesity can be attributed, in part, to the wide range of palatable foods that is available to us on a daily basis [1]. Eating palatable foods activates reward pathways in the brain (see [2] for a review), and enhancing the palatability of a food increases our intake of that food [3]. While the nature of palatability has been the subject of some debate [4], it is widely accepted that the sensory properties of food influence (if not determine) perceived palatability [5]. With this in mind, it is logical to propose that individual differences in sensory processing would affect the extent to which foods are perceived to be palatable, which would in turn influence food intake.

Sensory sensitivity is a ‘style’ of processing characterised by heightened sensitivity to environmental stimuli [6]. According to Dunn’s Model of Sensory Processing [6], sensory processing style results from an interaction between two continua: neurological threshold, and behavioural response. Individuals classified as being ‘sensory sensitive’ have a low neurological threshold (their *“neurons trigger more readily”* to sensory input ( [6], page 25), and respond passively in accordance with their threshold. Thus, a sensory sensitive person perceives stimuli at very low levels of sensory input, but (unlike an individual classified as ‘sensory avoidant’) does not attempt to counteract or avoid the strong sensory stimulation which overwhelms them. Sensory sensitivity is assessed using a self-report measure (e.g., in adults, the Adolescent/Adult Sensory Profile) which probes six sensory processing domains: taste/smell, movement, vision, touch, audition, and activity level. In the eating behaviour literature, sensory sensitivity has been associated with selective eating in children (e.g., [7]), and high sensitivity in the touch and taste/smell domains has been shown to predict low intake of fruit and vegetables [8]. It is thought that children with high sensory sensitivity avoid many fruits and vegetables due to their heightened perception of the varied tastes and textures of these foods, which makes eating them an aversive experience. It is proposed, here, that as well as making some foods more aversive due to their more perceptible sensory properties, sensory sensitivity may also make certain foods more desirable for the same reason. Specifically, it is predicted that sensory sensitive individuals might have an enhanced perception of palatability, due to a heightened sensitivity to the sensory properties of food. As food intake increases with palatability [4], it is possible that people who are sensory sensitive are more likely than people who are not sensory sensitive to (over-)consume highly-palatable foods.

This study sought to investigate the effect of sensory sensitivity on food intake. During the experiment, participants had access to a bowl of chocolate sweets as they completed questionnaires and a paper-based anagram task. Sensory sensitivity was assessed using a self-report measure, and chocolate intake during the experiment was compared between individuals classified as high in sensory sensitivity versus low-sensitivity. The original aim of this experiment was to investigate the effect of sensory sensitivity on food intake during stress; however as our manipulation failed to increase self-reported stress, the details of this aspect of the study are not reported in full here. Following reviewers’ requests, some details of the stress manipulation and mood measures are described briefly in the Method section. Information on the rationale behind the original aim, data analyses, and results can be provided by the corresponding author on request.

## Methods

### Participants

Forty female students from the University of Birmingham responded to an online advert for a ‘Mood, personality, and lifestyle’ experiment. Participants were aged between 18 and 25 years (*M* = 19.4; *SD* = 1.5), and all were psychology students participating for course credits. The participants’ body mass index (BMI) ranged from 16.3 to 33.2 (*M* = 22.3; *SD* = 3.7). The study was approved by the Ethics Committee of the University of Birmingham, and conformed to the principles set out in the Declaration of Helsinki.

### Measures

#### Sensory sensitivity

Sensory sensitivity was assessed using the Adult Sensory Profile [9], which is a 60-item questionnaire with high internal consistency (Cronbach’s α = .96, [10]). It assesses four sensory processing styles: ‘sensory sensitivity’, ‘sensory avoidance’, ‘sensation seeking’, and ‘low registration’, based on responses relating to the following aspects of processing: taste/smell, movement, vision, touch, audition, and activity level. Sensory sensitivity is assessed using 15 items, including “I become dizzy easily”, and “I don’t like certain food textures”, which participants respond to on a 5-point scale of how often they feel that way (from ‘almost never’ to ‘almost always’). The questionnaire given to participants included all 60 items, but only the responses to the sensory sensitivity items were analysed. The other items were included so that the random order of items in the original questionnaire could be retained, and to reduce the risk of the participants guessing the purpose of the study. Participants were classified as ‘high’ or ‘low’ in sensory sensitivity based on a median split of the scores.

#### Emotional eating and dietary restraint

Participants were also assessed on the ‘emotional eating’ and dietary restraint’ subscales of the Dutch Eating Behavior Questionnaire (DEBQ; [11]). This measure was included due to the original aim of investigating the stress-eating relationship. The DEBQ is a widely-used measure with high internal consistency and high validity. The 13-item emotional eating subscale assesses the extent to which people eat in response to experiencing negative mood, and includes questions such as “Do you have a desire to eat when you are depressed or discouraged?”, whilst the 10-item dietary restraint subscale consists of items such as “Do you try to eat less at mealtimes than you would like to eat?”. Participants responded to items using a 5-point scale ranging from ‘never’ (1) to ‘very often’ (5). Individual scores on all measures were used in the correlational analyses, and a median split was performed on each set of scores to classify participants as ‘emotional’ and ‘non-emotional’ eaters, and ‘restrained’ and ‘non-restrained’.

#### Anagram task and mood ratings

Due to the original aim to manipulate stress, participants completed an anagram task and completed a mood-rating questionnaire at two points during the experiment. The anagram task was designed to induce stress in half of the participants. All participants were given ten minutes to solve 15 anagrams; in the ‘non-stress’ group these were 4–6 letters long, whereas in the ‘stress’ group they were 6–8 letters long and only two were solvable. Participants rated their current feeling of 20 mood states, on 100 mm visual-analogue scales. Nineteen of the mood states were taken from the shortened version of the Profile of Mood States [12], and the final item was ‘stressed’.

### Procedure

Participants rated their mood, before being given ten minutes to complete the paper-based anagram task. The experimenter placed a bowl of ∼116 g of Galaxy Minstrels (Mars Inc., Slough, UK) on the table, telling participants to help themselves, and that they were an incentive to encourage participation. Minstrels are small milk chocolates encased in a crispy chocolate shell. Chocolate is a widely-liked food (e.g., [13]), and these sweets were considered particularly suitable for this experiment because they would not melt or crumble, so could be easily picked up and eaten by participants. The study advertisement included the statement *‘Free chocolate!’*, so it is likely that all of the participants liked chocolate. The bowls were weighed before and after the experiment to calculate the amount eaten by each participant. The experimenter left the room while the participant completed the anagram task, returning after ten minutes to remove the anagrams and give the participant the second set of mood ratings to complete. Finally, participants completed a questionnaire containing the items measuring sensory sensitivity, emotional eating, and dietary restraint, and height and weight measurements were taken. The Minstrels remained on the table until the end of the experiment.

### Data Analysis

A univariate analysis of variance (ANOVA) was used to compare the food intake of individuals with high and low sensory sensitivity, and correlational analyses were conducted to look for associations between food intake, sensory sensitivity, emotional eating, dietary restraint, and BMI. Further ANOVAs were performed to compare food intake between individuals classified as ‘emotional’ and ‘non-emotional’ eaters, and between ‘restrained’ and ‘non-restrained’ participants. Tests of normality were performed, and Spearman’s correlation coefficient was used to assess relationships involving non-normal data. All tests were carried out using SPSS Version 17.0 (Chicago, USA), and, for all analyses, statistical significance was determined with alpha set to.05.

## Results

**Figure 1:**
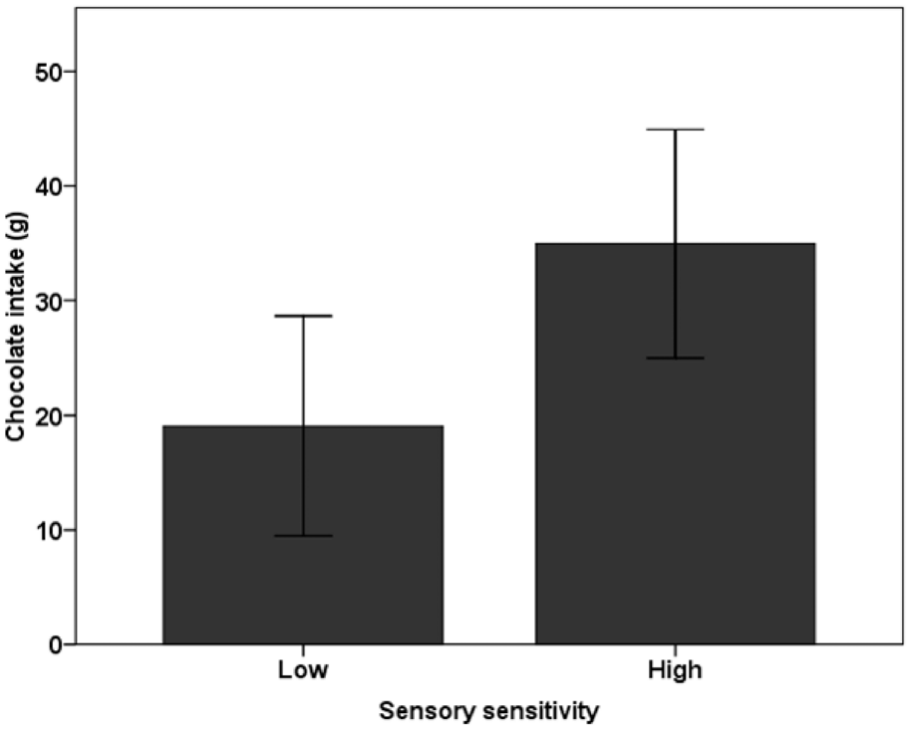
Effect of sensory sensitivity on food intake. Food intake of high sensory sensitive (*M = *35.0 g, *SD = *21.9 g) and low sensory sensitive (*M = *19.1 g, *SD = *19.9 g) individuals. Error bars represent 95% confidence intervals.

The ANOVA revealed a main effect of sensory sensitivity on food intake (*F*(1,38) = 5.73, *p* = .022; see Figure 1), with high-sensitivity participants eating significantly more chocolate (*M = *35.0 g, *SD = *21.9 g) than low-sensitivity participants (*M = *19.1 g, *SD = *19.9 g). In addition, a significant bivariate correlation was found between sensory sensitivity and emotional eating (*r* = .344, *p* = .030). No differences in food intake were found between emotional and non-emotional eaters (*F*(1,38) = 2.52, *p* = .120), or between restrained and non-restrained participants (*F*(1,38) = .264, *p* = .610), and no other significant correlations were found.

## Discussion

The finding that the high-sensitivity group ate more than the low-sensitivity group could be explained in a number of ways. To draw any firm conclusions about how sensory sensitivity affects food intake would go beyond the current findings; however, we believe that these data provide a good groundwork for further investigation of the role of sensory processing in overeating.

Sweet, high-fat foods are generally perceived as highly palatable (for review, see [14]), and it is thought that the sensory properties of chocolate, such as the creamy texture and sweet taste, along with the known psychoactive effects of chocolate, drive preference and cravings for this food (e.g., [15]). Given that sensory sensitivity is characterised by a heightened sensitivity to sensory input [6], and that the sensory properties of food influence how rewarding a food is [5], it is possible that people who are high in sensory sensitivity are more perceptive of the rewarding effects of palatable food. Indeed, functional neuroimaging data show individual differences in neural activation associated with food reward [16], and brain regions associated with reward such as the orbitofrontal cortex are also involved in taste and texture sensitivity [17]. Food intake is known to increase as palatability increases [3], so it is likely that if sensory sensitive individuals are more perceptive of palatability, they would eat more palatable foods in general.

In support of this conclusion, high sensitivity to reward has been previously associated with overeating and obesity. Davis and colleagues [18] found sensitivity to reward to be associated with overeating and a preference for sweet and high-fat foods, while another study [19] found positive associations with both BMI and food craving. The relationship between reward processing and food intake is not straightforward, however. Davis and Fox [20], for example, found that while sensitivity to reward was positively related to BMI in individuals classified as ‘normal’ or ‘overweight’ (BMI 18–30), within the ‘obese’ range (BMI>30) sensitivity to reward was negatively related to BMI. Indeed, ‘reward deficiency syndrome’, said to result from a *“biochemical inability to derive reward from ordinary, everyday activities”* ( [21] page 132) has been put forward as a possible cause of obesity [22]. It has been suggested that these discrepancies could be explained by different mechanisms underlying overeating in the overweight and ‘mildly obese’ compared to individuals who are morbidly obese [18]. As the majority (95%) of the participants in the present study had BMIs of less than 30, it could be argued that our findings complement the previously-reported data on sensitivity to reward and food intake [18], [20]. Specifically, our data could indicate that the relationship between sensitivity to reward and overeating [18] is mediated by sensory sensitivity, but only in individuals within the 18–30 BMI range.

The relationship between sensory sensitivity and emotional eating, which was revealed by an exploratory analysis of these two variables, indicates that as sensory sensitivity increases, so does one’s tendency to eat in response to negative mood. It is possible that sensory sensitive individuals either experience negative emotions differently, or use different strategies (e.g., overeating) to deal with them. Indeed, sensory processing style has been shown to influence how individuals experience and cope with anxiety [23], so it is possible that emotional eating is one of the ways in which sensory sensitive individuals differ from others during stressful or anxiety-provoking periods.

There are, however, some potential limitations to this study. The first is that the participants may not have been equally hungry at the time of the experiment. As the participants were not instructed to arrive in a hungry or satiated state, and testing times were at various times throughout the day, hunger may have influenced participants’ intake of chocolate. This is not perceived as a threat to the current findings, but is something that should be addressed in replications of this study. A further improvement to the methodology would be to take an explicit measure of liking for chocolate. Although our study advertisement indicated that participation would involve eating chocolate, so it was assumed that all of the participants did like chocolate, it may have been useful to include liking of chocolate as a covariate in our analyses. A final consideration is that, due to the original aim to manipulate stress, participants were not all doing exactly the same task. Again, this is not considered a threat to the reported findings, because the numbers of ‘low-’ and ‘high-’ sensitivity participants in each of the ‘stress’ and ‘non-stress’ groups were not significantly different; however this difference should be taken into account when considering the reported effects.

The results of this study strongly suggest that food intake is influenced by sensory sensitivity; the reasons for this, however, can only speculated from the current data. In the context of previous research, it is suggested that the rewarding effect of eating palatable foods is intensified in individuals who are sensory sensitive, due to a heightened sensitivity to the sensory properties of food, which influence palatability.
